# Plasma Retinol Levels and High-Sensitivity C-Reactive Protein in Prepubertal Children

**DOI:** 10.3390/nu10091257

**Published:** 2018-09-07

**Authors:** Olaya de Dios, Pilar Navarro, Henar Ortega-Senovilla, Leticia Herrero, Teresa Gavela-Pérez, Leandro Soriano-Guillen, Miguel A. Lasunción, Carmen Garcés

**Affiliations:** 1 Lipid Laboratory, IIS-Fundación Jiménez Díaz, UAM, 28040 Madrid, Spain; olaya.dios@quironsalud.es (O.d.D.); mpilar_ns@hotmail.com (P.N.); leticia.herrerot@quironsalud.es (L.H.); 2 Faculties of Pharmacy and Medicine, Universidad San Pablo-CEU, 28925 Madrid, Spain; henar@ceu.es; 3 Department of Pediatrics, IIS-Fundación Jiménez Díaz, UAM, 28040 Madrid, Spain; TGavela@quironsalud.es (T.G.-P.); LSoriano@fjd.es (L.S.-G.); 4 Servicio de Bioquímica-Investigación, Hospital Universitario Ramón y Cajal, 28034 Madrid, Spain; miguel.a.lasuncion@hrc.es

**Keywords:** C-reactive protein, hs-CRP concentrations, fat-soluble plasma antioxidants, plasma retinol concentrations, prepubertal children

## Abstract

The relationship between C-reactive protein (CRP) levels and plasma antioxidants has been established in adults. However, the association has been rarely investigated in healthy children. Thus, we examined the cross-sectional association of high-sensitivity CRP (hs-CRP) levels with fat-soluble plasma antioxidant concentrations in a cohort of healthy prepubertal children. We determined hs-CRP levels in 543 healthy six–eight-year-old children using a high-sensitivity CRP enzyme-linked immuno sorbent assay (ELISA) kit. The plasma concentrations of lipids, apolipoproteins and lipid-soluble antioxidants (α-tocopherol, γ-tocopherol, lycopene, α-carotene, β-carotene and retinol) were determined using standardized methods. Pearson correlation analysis showed significant correlations between plasma hs-CRP and α-carotene and retinol concentrations. After adjusting by sex, body mass index (BMI) and lipid levels, only the association with retinol remains significant, with children in the highest hs-CRP tertile group (hs-CRP ≥ 0.60 mg/dL) showing significantly lower levels of retinol than those from the tertiles 1 and 2. A stepwise linear regression selected retinol, BMI, apo A-I and sex as predictors of hs-CRP levels, in a model explaining 19.2% of the variability of hs-CRP. In conclusion, in healthy prepubertal children, after adjusting by sex, BMI and lipid levels, hs-CRP concentrations were highly associated with plasma retinol, which is transported in blood bound to retinol-binding protein but were not associated with the lipoprotein-bound antioxidants.

## 1. Introduction

C-reactive protein (CRP) is an acute-phase inflammatory protein synthesized in the liver through the stimulation of interleukin-6 that has been extensively studied as a marker of the subclinical inflammation associated with obesity, metabolic syndrome, diabetes and cardiovascular disease [1].

It is known that oxidative stress has pro-inflammatory effects, and an important role in this association has been attributed to CRP [2]. Plasma antioxidants seem to decline during the “acute-phase response” in the inflammatory process that is associated with the increase of inflammatory markers, such as CRP [3]. However, the association of these biomarkers with reduced antioxidant levels in low-grade inflammation remains under study. A meta-analysis of randomized controlled trials analyzing the effect of vitamin E supplementation suggests that supplementation with either α-tocopherol or γ-tocopherol reduces blood CRP concentrations [4]. Schwab et al. [5] suggested that this association was found only with vitamin E in combination with the intake of other antioxidants. Epidemiological studies to date analyzing the association of dietary antioxidants and serum vitamins with CRP levels in adults have produced diverse evidence. In some studies, CRP concentrations have been related to lower levels of carotenoids, vitamin E and vitamin A levels [6,7,8,9,10,11,12], while other investigations have failed to find an association of CRP levels with vitamin E [13] or α-tocopherol [8], for example. Race differences in the relation of vitamins A, E and β-carotene with CRP have been reported [14].

Studies analyzing the association between CRP and antioxidant levels in children are scarce and it has been mostly investigated in sick children. The association between CRP and vitamin A, for example, has been analyzed in children with an infectious disease [15], night blindness [16] or obesity [17,18]. To our knowledge, no cross-sectional studies analyzing the association of CRP with vitamin levels have been performed in healthy children in developed countries.

In our study, we examined the association of hs-CRP levels with plasma antioxidant (α-tocopherol, γ-tocopherol, lycopene, α-carotene, β-carotene and retinol) concentrations in a cohort of healthy prepubertal children in Spain.

## 2. Materials and Methods 

### 2.1. Subjects

This is a sub-study of the “Four Provinces Study”, a broad cross-sectional study designed to analyze cardiovascular risk factors in Spanish schoolchildren. Our sample comprises 543 6–8-year-old children (289 girls and 254 boys). All children included in the study were free of any endocrine, metabolic, hepatic or renal disorder. Parents were required to sign a written consent form allowing their children to participate. The study protocol was approved by the Ethics Committee of Clinical Investigation of the Fundación Jiménez Díaz. The investigation fulfills the principles contained in the Declaration of Helsinki and subsequent reviews, as well as the prevailing Spanish legislation on clinical research in human subjects.

### 2.2. Data Collection

A team consisting of one physician and several nurses was in charge of blood extractions and physical measurements.

### 2.3. Anthropometric Variables

Measurements (weight and height) were taken with children barefoot and wearing light clothing. Weight was determined to the nearest 0.1 kg and height was measured to the nearest 0.1 cm. Body mass index (BMI) (weight in kilograms divided by height in meters squared, kg/m^2^) was calculated from these parameters.

### 2.4. Biochemical Data

Blood samples were obtained early in the morning after a 12-h fasting period using venipuncture. Samples were kept on ice and sent to the laboratory for analysis. Samples were centrifuged at 1500× *g* at 4 °C for 20 min. Once centrifuged, fractions were separated and frozen at −70 °C for future analyses.

Cholesterol and triglycerides were measured enzymatically (Menarini) with a RA-1000 Autoanalyzer (Technicon, Luton, UK). High-density lipoprotein (HDL) cholesterol was measured after the precipitation of apo B-containing lipoproteins with phosphotungstic acid and Mg (Boehringer Mannheim, Baden-Wurttemberg, Germany). Low-density lipoprotein (LDL) cholesterol was calculated according to Friedewald´s formula. Plasma apo A-I and apo B concentrations were measured using immunonephelometry (Dade Berhing, Deerfield, IL, USA). The interassay coefficients of variation were: cholesterol, 1.4%; triglyceride, 1.7%; apo A-I, 1.55% and apo B, 4.8%. 

Plasma α-tocopherol, γ-tocopherol, lycopene, α-carotene, β-carotene and retinol were measured using isocratic high-performance liquid chromatography-based methods (HPLC) (Beckman System Gold High Performance Liquid Chromatograph, NM, USA) based on the method described by H. Ortega et al. [19]. Retinol acetate and tocopherol acetate were used as internal standards. The standard reference material SRM 968c from the National Institute of Standards and Technology was used as a control. The interassay coefficients of variation were: α-tocopherol, 5.0 %; γ-tocopherol, 12.5%; lycopene, 5.5%; α-carotene, 8.8%; β-carotene, 7.8% and retinol, 4.4%. 

CRP levels were measured using a high-sensitivity CRP ELISA kit (SK00080-02, Aviscera Bioscience, Inc., Santa Clara, CA, USA). The sensitivity of the assay was 0.15 mg/L.

### 2.5. Statistical Analysis

Children with hs-CRP levels equal to or above 10 mg/L were excluded from the analysis. The normality of distributions of the variables was analyzed using the Kolmogorov-Smirnov test. Variables that were not normally distributed (hs-CRP, BMI, triglyceride, α-tocopherol, lycopene, α-carotene and β-carotene) were log-transformed to normality prior to the analyses. Mean values are shown as means ± SD. We used a t-test to compare the study variables by sex. Pearson correlation coefficients were calculated to evaluate the correlations between hs-CRP and lipid variables and fat-soluble antioxidants. To ascertain the independent predictors of plasma hs-CRP levels, a stepwise lineal regression analysis was performed. For this, the independent variables were selected among BMI, plasma lipid and apolipoprotein concentrations and fat-soluble antioxidant levels. An analysis of variance (ANOVA) was used to compare the means of the antioxidant levels between groups depending on different hs-CRP levels (hs-CRP tertiles), after adjusting for possible confounding factors (sex, age, BMI, lipid and apolipoprotein levels). Post-hoc multiple comparisons were performed using the Tukey’s test. A statistical analysis was performed using the SPSS software package, version 21.0 (SPSS, Inc., Chicago, IL, USA).

## 3. Results

The average age of the children in our study was 6.7 years, similar in both genders. Mean BMI and biochemical variables by gender are shown in Table 1. No statistically significant differences in BMI, plasma lipid and apolipoprotein levels or hs-CRP concentrations between boys and girls were found (Table 1). 

Pearson´s correlation coefficients between hs-CRP levels and BMI and plasma antioxidants, and between these vitamins and lipid and apolipoprotein concentrations are shown in Table 2. There were significant correlations between hs-CRP and BMI, β-carotene and retinol concentrations. As previously described in our population, hs-CRP also correlated negatively with HDL cholesterol and apo A-I, and positively with triglycerides (TG) concentrations [20]. As expected, plasma concentrations of fat-soluble antioxidants were significantly correlated with plasma lipids. α-tocopherol was correlated with all the lipid variables; the highest correlation coefficients being found with total cholesterol and apo B concentrations. γ-tocopherol was correlated with total cholesterol, LDL cholesterol and apo B. Lycopene and carotenes positively correlated with total cholesterol, lipoprotein-cholesterol and apolipoproteins levels, but not with triglyceride. Plasma retinol concentration showed a positive correlation with HDL cholesterol, apo A-I and apo B (Table 2).

**Figure 1:**
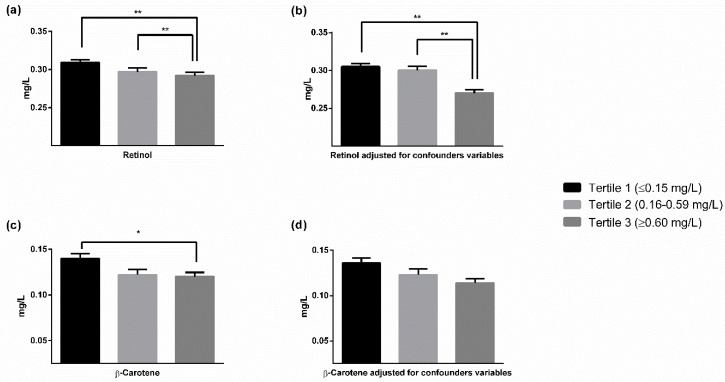
(**a**) Plasma levels of retinol (mg/L) according to hs-CRP tertiles unadjusted; (**b**) plasma levels of retinol (mg/L) according to hs-CRP tertiles adjusted for confounders variables; (**c**) plasma levels of β-Carotene (mg/L) according to hs-CRP tertiles unadjusted; (**d**) plasma levels of β-Carotene (mg/L) according to hs-CRP tertiles adjusted for confounder variables. * *p* < 0.01, ** *p* < 0.001.

For the analysis, the sample was divided into tertiles of hs-CRP levels. Children in the highest hs-CRP tertile group (hs-CRP ≥ 0.60 mg/dL) presented significantly lower levels of retinol and β-carotene than those from tertiles 1 and 2 (Figure 1). These differences only remain significant when adjusting for BMI and lipid levels for plasma retinol concentrations (0.270 ± 0.063 in tertile 3 versus 0.300 ± 0.065 in tertile 2 and 0.305 ± 0.056 in tertile 1, *p* < 0.001). 

We further analyzed the independent determinants of hs-CRP plasma concentrations using a stepwise linear regression. The selected variables along with the standardized coefficients (**β**) and the p values for each predictor, and the *R*^2^ values of the model are given in Table 3. Retinol, BMI, apo A-I and sex were selected as predictors in a model explaining as much as 19.2 % of the variability of hs-CRP. The magnitude of the contribution of both retinol and BMI was higher than the contribution of sex or apo A-I (Table 3).

## 4. Discussion

This study describes the relationship between the plasma concentration of fat-soluble antioxidants and hs-CRP levels in a healthy cohort of prepubertal children where the effect of sex hormones, alcohol consumption and the smoking habit can be avoided. We have identified a strong association between plasma retinol levels and hs-CRP concentrations in these children, independently of BMI and lipid levels, with our results showing significantly lower plasma retinol levels in children in the highest hs-CRP concentration tertile. 

There are few published reports on the analysis of the relationship between plasma fat-soluble antioxidants and hs-CRP concentrations in prepuberal children in developed countries. Studies analyzing this association in children have focused mainly on sick children [15,16,21], and, to our knowledge, this association has not been previously described in healthy children in our area. Thus, our study adds to the literature that has investigated this association mostly in adults, showing a clear association between hs-CRP and plasma retinol levels in healthy children in a Mediterranean country, such as Spain.

In our population, even though β-carotene concentrations also appear to be related to hs-CRP levels, after adjusting by BMI and lipid levels, retinol concentration emerges as the only fat-soluble antioxidant related to hs-CRP levels in our children. Vitamin A deficiency has been associated with CRP levels among preschool children with night blindness [16] and low concentrations of vitamin A has been described in overweight and obese Mexican children [22] but, to our knowledge, no cross-sectional studies have investigated, in healthy Caucasian children, the association of hs-CRP with retinol or any of the other lipid-soluble antioxidants we have analyzed. 

Interestingly, we found an association between hs-CRP levels and the plasma concentration of retinol, which is transported in blood bound to retinol-binding protein, not to lipoproteins, and we failed to find any association of hs-CRP levels with the concentrations of any of the lipoprotein-bound fat-soluble antioxidants analyzed. Since retinol concentration is tightly regulated by the retinol-binding protein (RBP) [22], we hypothesized that RBP could have a role in the association between retinol and CRP levels observed in children. A correlation between RBP4 and CRP levels has been described in obese children [23]. Further studies are needed to clarify this hypothesis. 

The complex interrelatedness between CRP and oxidized low-density lipoproteins has been reviewed elsewhere [24]. The lack of an association between hs-CRP levels and the lipoprotein-bound fat-soluble antioxidant concentrations in children, which has been extensively reported in adults [6,7,9,12], may be due to the fact that the inflammatory status related to LDL oxidation is evident in adults but not in children. Thus, lipoprotein-bound antioxidants may play a role regarding LDL oxidation susceptibility in adults but not in children. The association between vitamin A and lower CRP levels has also been reported in the study by Garcia et al. [22] analyzing Mexican school-aged children. Similar to our findings for β-carotene, in this study the association between CRP levels and vitamin E disappears when considering the vitamin E/lipids ratio [22]. Further studies are required to clarify these aspects.

In our population, we have previously described a significant association between a greater fruit and vegetable intake and lower hs-CRP levels [25], which we linked to the high fiber and antioxidant contents related to this vegetable and fruit intake. In addition to the consistent association between hs-CRP concentrations and fiber intake [10,26], the contribution of vitamin intake associated with vegetable and fruit consumption has also been accepted. A relationship between plasma hs-CRP concentration and vitamin A and vitamin E intake has been reported in our children [25]. Here, we confirmed the association between hs-CRP and retinol plasma concentrations but failed to demonstrate its association with α-tocopherol or γ-tocopherol.

As previously described in our children, we did not find any statistically significant correlation between plasma fat-soluble antioxidants and the major nutrients or vitamins consumed [20]. As regards retinol, other studies fail to find any association between dietary intake and plasma concentration in well-nourished populations [27]. Regarding the influence of diet on serum tocopherol, studies in adult populations have provided conflicting results. Some authors have reported a small association of dietary intake of vitamin E with serum tocopherol concentration [28], while others found no significant association [27,29,30].

## 5. Conclusions

Our study in six–eight-year-old children reports an important association between hs-CRP concentrations and plasma retinol which is transported in blood bound to retinol-binding protein, and fails to find any association between hs-CRP levels and the lipoprotein-bound fat-soluble antioxidants. These findings suggest an important role of retinol in preventing inflammation in early age stages.

## Figures and Tables

**Table 1:** Body mass index (BMI) and plasma biochemical variables (mean ± SD) in prepubertal children by sex.

	Boys (*n* = 254)	Girls (*n* = 287)
BMI	17.2 ± 2.6	17.2 ± 2.7
hs-CRP (mg/L) ^a^	0.90 ± 1.52	0.99 ± 1.71
Total cholesterol (mg/dL)	183.9 ± 27.1	183.5 ± 29.1
Triglycerides (mg/dL) ^a^	70.7 ± 27.9	72.4 ± 24.0
HDL cholesterol (mg/dL)	59.4 ± 13.1	58.5 ± 13.5
LDL cholesterol (mg/dL)	110.3 ± 26.6	110.5 ± 26.9
Apo A-I (mg/dL)	136.0 ± 18.4	133.6 ± 18.1
Apo B (mg/dL)	70.2 ± 14.2	70.8 ± 15.0
α-tocopherol (mg/L) ^a^	9.07 ± 1.61	9.37 ± 1.74 *
γ-tocopherol (mg/L)	0.95 ± 0.40	0.95 ± 0.41
Lycopene (mg/L) ^a^	0.187 ± 0.129	0.183 ± 0.129
α-carotene (mg/L) ^a^	0.033 ± 0.023	0.031 ± 0.020
β-carotene (mg/L) ^a^	0.129 ± 0.074	0.123 ± 0.066
Retinol (mg/L)	0.29 ± 0.06	0.30 ± 0.064

^a^ Log-transformed variables, * *p* < 0.05. hs-CRP: High sensitivity CRP, HDL: High-density lipoprotein; LDL: Low-density lipoprotein.

**Table 2:** Pearson correlation coefficients between plasma concentrations of fat-soluble antioxidants and lipids, apolipoproteins, high sensitivity-CRP and BMI in children.

	hs-CRP ^a^	BMI ^a^	Triglycerides ^a^	Cholesterol	LDL-C	HDL-C	Apo B	Apo A-I
**α-Tocopherol** ^a^	−0.013	−0.012	0.134 **	0.421 ***	0.296 ***	0.250 ***	0.459 ***	0.256 ***
**γ-Tocopherol**	0.033	0.082	0.047	0.136 **	0.104 *	0.058	0.141 ***	0.059
**Lycopene** ^a^	−0.073	0.065	−0.064	0.119 **	0.062	0.149 ***	0.085 *	0.216 ***
**α-Carotene** ^a^	−0.066	−0.085	−0.074	0.151 ***	0.112 **	0.129 **	0.144 ***	0.133 **
**β-Carotene** ^a^	−0.144 ***	−0.074	−0.065	0.219 ***	0.172 ***	0.147 ***	0.226 ***	0.173 ***
**Retinol**	−0.280 ***	0.192 ***	0.063	0.071	−0.030	0.167 ***	0.125 **	0.214 ***

^a^ Log-transformed skewed data. * *p* < 0.05, ** *p* < 0.01, *** *p* < 0.001.

**Table 3:** Results of linear regression model to identify independent determinants of hs-CRP plasma concentrations.

Base Line Variable	β	*p*-Value
Retinol	−0.315	<0.001
BMI *	0.287	<0.001
Apo A-I	−0.138	0.002
Gender	0.113	0.009

*R*^2^: 19.2; * Log-transformed data.

## References

[1] Kaptoge S., Di Angelantonio E., Lowe G., Pepys M.B., Thompson S.G., Collins R., Danesh J., Tipping R.W., Ford C.E., Pressel S.L. (2010). C-reactive protein concentration and risk of coronary heart disease, stroke, and mortality: An individual participant meta-analysis. Lancet.

[2] Kobayashi S., Inoue N., Ohashi Y., Terashima M., Matsui K., Mori T., Fujita H., Awano K., Kobayashi K., Azumi H. (2003). Interaction of oxidative stress and inflammatory response in coronary plaque instability: Important role of C-reactive protein. Arterioscler. Thromb. Vasc. Biol..

[3] Louw J.A., Werbeck A., Louw M.E.J., Kotze T.J.V.W., Cooper R., Labadarios D. (1992). Blood vitamin concentrations during the acute-phase response. Crit. Care Med..

[4] Saboori S., Shab-Bidar S., Speakman J.R., Yousefi Rad E., Djafarian K. (2015). Effect of Vitamin E supplementation on serum C-reactive protein level: A meta-analysis of randomized controlled trials. Eur. J. Clin. Nutr..

[5] Schwab S., Zierer A., Schneider A., Heier M., Koenig W., Kastenmüller G., Waldenberger M., Peters A., Thorand B. (2015). Vitamin e supplementation is associated with lower levels of C-reactive protein only in higher dosages and combined with other antioxidants: The Cooperative Health Research in the Region of Augsburg (KORA) F4 study. Br. J. Nutr..

[6] Erlinger T.P., Guallar E., Miller E.R., Stolzenberg-Solomon R., Appel L.J. (2001). Relationship between systemic markers of inflammation and serum beta-carotene levels. Arch. Intern. Med..

[7] Kritchevsky S.B., Bush A.J., Pahor M., Gross M.D. (2000). Serum carotenoids and markers of inflammation in nonsmokers. Am. J. Epidemiol..

[8] Il’Yasova D., Ivanova A., Morrow J.D., Cesari M., Pahor M. (2008). Correlation between two markers of inflammation, serum C-reactive protein and interleukin 6, and indices of oxidative stress in patients with high risk of cardiovascular disease. Biomarkers.

[9] Wood A.D., Strachan A.A., Thies F., Aucott L.S., Reid D.M., Hardcastle A.C., Mavroeidi A., Simpson W.G., Duthie G.G., Macdonald H.M. (2014). Patterns of dietary intake and serum carotenoid and tocopherol status are associated with biomarkers of chronic low-grade systemic inflammation and cardiovascular risk. Br. J. Nutr..

[10] Oliveira A., Rodríguez-Artalejo F., Lopes C. (2009). The association of fruits, vegetables, antioxidant vitamins and fibre intake with high-sensitivity C-reactive protein: Sex and body mass index interactions. Eur. J. Clin. Nutr..

[11] Yang M., Chung S.-J., Floegel A., Song W.O., Koo S.I., Chun O.K. (2013). Dietary antioxidant capacity is associated with improved serum antioxidant status and decreased serum C-reactive protein and plasma homocysteine concentrations. Eur. J. Nutr..

[12] Ford E.S., Liu S., Mannino D.M., Giles W.H., Smith S.J. (2003). C-reactive protein concentration and concentrations of blood vitamins, carotenoids, and selenium among United States adults. Eur. J. Clin. Nutr..

[13] Cheng H.G., Alshaarawy O., Cantave M.D., Anthony J.C. (2016). Inverse association linking serum levels of potential antioxidant vitamins with C-reactive protein levels using a novel analytical approach. Br. J. Nutr..

[14] Suarez E.C., Schramm-Sapyta N.L. (2014). Race differences in the relation of vitamins A, C, E, and β-carotene to metabolic and inflammatory biomarkers. Nutr. Res..

[15] Mitra A.K., Alvarez J.O., Wahed M.A., Fuchs G.J., Stephensen C.B. (1998). Predictors of serum retinol in children with shigellosis. Am. J. Clin. Nutr..

[16] Maqsood M., Dancheck B., Gamble M.V., Palafox N.A., Ricks M.O., Briand K., Semba R.D. (2004). Vitamin A deficiency and inflammatory markers among preschool children in the Republic of the Marshall Islands. Nutr. J..

[17] Stenzel A.P., Carvalho R., Jesus P., Bull A., Pereira S., Saboya C., Ramalho A. (2018). Serum antioxidant associations with metabolic characteristics in metabolically healthy and unhealthy adolescents with severe obesity: An observational study. Nutrients.

[18] Yang C., Chen J., Liu Z., Yun C., Li Y., Piao J., Yang X. (2015). Association of vitamin a status with overnutrition in children and adolescents. Int. J. Environ. Res. Public Health.

[19] Ortega H., Coperías J.L., Castilla P., Gómez-Coronado D., Lasunción M.A. (2004). Liquid chromatographic method for the simultaneous determination of different lipid-soluble antioxidants in human plasma and low-density lipoproteins. J. Chromatogr. B Anal. Technol. Biomed. Life Sci..

[20] Ortega H., Castilla P., Gómez-Coronado D., Garcés C., Benavente M., Rodríguez-Artalejo F., De Oya M., Lasunción M.A. (2005). Influence of apolipoprotein E genotype on fat-soluble plasma antioxidants in Spanish children. Am. J. Clin. Nutr..

[21] Beisel W.R. (1998). Infection-induced depression of serum retinol—A component of the acute phase response or a consequence?. Am. J. Clin. Nutr..

[22] García O.P., Ronquillo D., del Carmen Caamaño M., Martínez G., Camacho M., López V., Rosado J.L. (2013). Zinc, iron and vitamins A, C and E are associated with obesity, inflammation, lipid profile and insulin resistance in Mexican school-aged children. Nutrients.

[23] Balagopal P., Graham T.E., Kahn B.B., Altomare A., Funanage V., George D. (2007). Reduction of elevated serum retinol binding protein in obese children by lifestyle intervention: Association with subclinical inflammation. J. Clin. Endocrinol. Metab..

[24] Obradovic M.M., Trpkovic A., Bajic V., Soskic S., Jovanovic A., Stanimirovic J., Panic M., Isenovic E.R. (2015). Interrelatedness between C-reactive protein and oxidized low-density lipoprotein. Clin. Chem. Lab. Med..

[25] Navarro P., de Dios O., Jois A., Gavela-Pérez T., Gorgojo L., Martín-Moreno J.M., Soriano-Guillen L., Garcés C. (2017). Vegetable and fruit intakes are associated with hs-CRP levels in pre-pubertal girls. Nutrients.

[26] King D.E., Egan B.M., Woolson R.F., Mainous A.G., Al-Solaiman Y., Jesri A. (2007). Effect of a high-fiber diet vs a fiber-supplemented diet on C-reactive protein level. Arch. Intern. Med..

[27] Hebert J.R., Hurley T.G., Hsieh J., Rogers E., Stoddard A.M., Sorensen G., Nicolosi R.J. (1994). Determinants of plasma vitamins and lipids: The working well study. Am. J. Epidemiol..

[28] White E., Kristal A.R., Shikany J.M., Wilson A.C., Chen C., Mares-Perlman J.A., Masaki K.H., Caan B.J. (2001). Correlates of serum α- and γ-tocopherol in the women’s health initiative. Ann. Epidemiol..

[29] Ascherio A., Stampfer M.J., Colditz G.A., Rimm E.B., Litin L., Willett W.C. (1992). Correlations of Vitamin A and E Intakes with the Plasma Concentrations of Carotenoids and Tocopherols among American Men and Women. J. Nutr..

[30] Sinha R., Patterson B.H., Mangels A.R., Levander O.A., Gibson T., Taylor P.R., Block G. (1993). Determinants of plasma vitamin E in healthy males. Cancer Epidemiol. Biomarkers Prev..

